# Extracorporeal lung support in trauma patients with severe chest injury and acute lung failure: a 10-year institutional experience

**DOI:** 10.1186/cc12782

**Published:** 2013-06-20

**Authors:** Michael Ried, Thomas Bein, Alois Philipp, Thomas Müller, Bernhard Graf, Christof Schmid, David Zonies, Claudius Diez, Hans-Stefan Hofmann

**Affiliations:** 1 Department of Thoracic Surgery, University Medical Center Regensburg, Franz-Josef-Strauss-Allee 11, 93053 Regensburg, Germany; 2 Department of Anesthesiology, University Medical Center Regensburg, Franz-Josef-Strauss-Allee 11, 93053 Regensburg, Germany; 3 Department of Cardiothoracic Surgery, University Medical Center Regensburg, Franz-Josef-Strauss-Allee 11, 93053 Regensburg, Germany; 4 Department of Internal Medicine II, University Medical Center Regensburg, Franz-Josef-Strauss-Allee 11, 93053 Regensburg, Germany; 5 US Army Landstuhl Regional Medical Center, Dr. Hitzelberger Strasse, 66849 Landstuhl/Kirchberg, Germany

**Keywords:** Trauma, Chest injury, Acute lung failure, Extracorporeal lung support, Extracorporeal membrane oxygenation

## Abstract

**Introduction:**

Severe trauma with concomitant chest injury is frequently associated with acute lung failure (ALF). This report summarizes our experience with extracorporeal lung support (ELS) in thoracic trauma patients treated at the University Medical Center Regensburg.

**Methods:**

A retrospective, observational analysis of prospectively collected data (Regensburg ECMO Registry database) was performed for all consecutive trauma patients with acute pulmonary failure requiring ELS during a 10-year interval.

**Results:**

Between April 2002 and April 2012, 52 patients (49 male, three female) with severe thoracic trauma and ALF refractory to conventional therapy required ELS. The mean age was 32 ± 14 years (range, 16 to 72 years). Major traffic accident (73%) was the most common trauma, followed by blast injury (17%), deep fall (8%) and blunt trauma (2%). The mean Injury Severity Score was 58.9 ± 10.5, the mean lung injury score was 3.3 ± 0.6 and the Sequential Organ Failure Assessment score was 10.5 ± 3. Twenty-six patients required pumpless extracorporeal lung assist (PECLA) and 26 patients required veno-venous extracorporeal membrane oxygenation (vv-ECMO) for primary post-traumatic respiratory failure. The mean time to ELS support was 5.2 ± 7.7 days (range, <24 hours to 38 days) and the mean ELS duration was 6.9 ± 3.6 days (range, <24 hours to 19 days). In 24 cases (48%) ELS implantation was performed in an external facility, and cannulation was done percutaneously by Seldinger's technique in 98% of patients. Cannula-related complications occurred in 15% of patients (PECLA, 19% (*n *= 5); vv-ECMO, 12% (*n *= 3)). Surgery was performed in 44 patients, with 16 patients under ELS prevention. Eight patients (15%) died during ELS support and three patients (6%) died after ELS weaning. The overall survival rate was 79% compared with the proposed Injury Severity Score-related mortality (59%).

**Conclusion:**

Pumpless and pump-driven ELS systems are an excellent treatment option in severe thoracic trauma patients with ALF and facilitate survival in an experienced trauma center with an interdisciplinary treatment approach. We encourage the use of vv-ECMO due to reduced complication rates, better oxygenation and best short-term outcome.

## Introduction

Severe multiple trauma is often associated with traumatic lung injury and presents with a wide spectrum of severity. In recent databases, patients with multiple trauma are reported to suffer from associated chest injury in nearly 50% of cases [1]. However, only a minority of patients develop post-traumatic respiratory insufficiency that mandates intubation and mechanical ventilation [2]. Generally, the management of both blunt and penetrating thoracic injuries is supportive and should aim to minimize the systemic inflammatory response syndrome and its progression to acute lung failure (ALF) or acute respiratory distress syndrome (ARDS) [3]. Nevertheless, ALF and ARDS are severe and common complications of major thoracic trauma [4]. ALF is characterized by a life-threatening impairment of the pulmonary gas exchange, resulting in hypoxia, hypercapnia and respiratory acidosis [5]. But despite recent advantages in critical management, severe thoracic injuries with ALF or cardiopulmonary insufficiency present a challenge and are still associated with high morbidity and mortality [6,7]. Conventional mechanical ventilation strategies are the mainstay of treatment for ALF associated with thoracic trauma [8]. But trauma patients with critical respiratory insufficiency (life-threatening hypoxemia and/or severe hypercapnia/acidosis) refractory to optimized conventional treatment strategies may finally benefit from a rescue extracorporeal gas exchange [9].

Extracorporeal lung support (ELS) devices such as pumpless extracorporeal lung assist (PECLA) or extracorporeal membrane oxygenation (ECMO) may be used as a temporary replacement for the damaged lungs in order to provide sufficient ventilation, oxygenation, improvement of hypercapnia and time for recovery of the lungs, after all treatment options including invasive ventilation have failed [3,6,10,11]. Nevertheless, the impact of ECMO support in patients with severe pulmonary failure due to traumatic life-threatening injuries remains controversial especially due to the risk of bleeding complications, and its application is limited to a few experienced centers [2,12].

This institutional report summarizes prospectively collected data and describes our interdisciplinary experience with ELS including PECLA and veno-venous extracorporeal membrane oxygenation (vv-ECMO) in severe thoracic trauma patients with ALF treated at the University Medical Center Regensburg during a 10-year interval.

## Patients and methods

### Patients and indications

This was a retrospective analysis of prospectively collected data from the Regensburg ECMO Registry. Between April 2002 and April 2012 a total of 52 patients with severe trauma and concomitant chest injury causing ALF with the need for ELS were interdisciplinary treated at the University Medical Center Regensburg and included in this study sample. The study had a retrospective design and was approved by the local ethics committee of the University Medical Center Regensburg. The requirement of individual patient consent was waived because of the study's retrospective design and data collection from routine care. Our Institutional Review Board waived the necessity of approval for the data report.

We describe our institutional experience with both pumpless and pump-driven devices in severe trauma patients. Patient characteristics, laboratory data, ventilation parameters, ELS therapy, surgical procedures and clinical outcome data were recorded and evaluated. Severe post-traumatic ALF was defined by partial pressure of arterial oxygen (PaO_2_)/fraction of inspired oxygen (FiO_2_) ratio <80 mmHg, a maximum positive end-expiratory pressure (18 cmH_2_O) and persistent respiratory acidosis (pH <7.25) despite optimized mechanical ventilation and optimization of conservative treatment options. Before implantation of an ELS device, a trial to improve the pulmonary gas exchange was conducted following the institutional protocol, including lung recruitment maneuvers, inhalation of vasodilators (nitric oxide, prostacyclin), kinetic therapy (prone positioning) and high-frequency oscillatory ventilation depending on the patient's status [13]. ALF was treated either with PECLA or with vv-ECMO. Three patients with persisting cardiopulmonary failure despite improved gas exchange were switched to secondary veno-arterial ECMO after primary ELS (1× PECLA, 2× vv-ECMO). During the study period four trauma patients with cardiopulmonary failure required primary implantation of a veno-arterial ECMO for hemodynamic stabilization. These patients were excluded from this analysis. The primary endpoint was survival to discharge from our hospital.

### Techniques of extracorporeal lung support

Until 2008, patients with ALF were treated with PECLA (Novalung GmbH, Heilbronn, Germany) developed in 1996 by Philipp and colleagues [14]. Technical data and the implantation technique have been described in detail by our institution [11,15]. In principle, the PECLA is an artificial arterio-venous shunt with an interposed extracorporeal membrane oxygenator without the need of a pump [16]. The prerequisite is normal left ventricular function and an absence of a distinctive peripheral arteriosclerosis. The purpose of this method is the effective elimination of carbon dioxide with a modest increase in arterial oxygenation and rapid normalization of respiratory acidosis.

**Figure 1:**
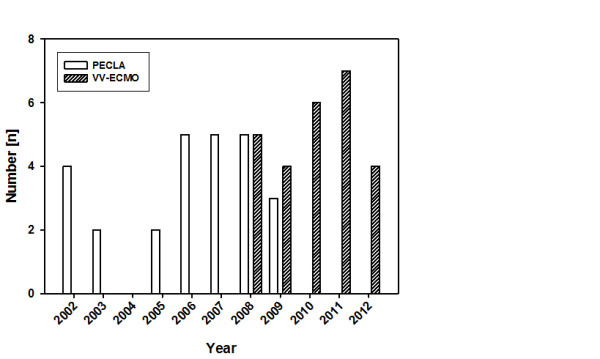
**Distribution of both extracorporeal lung support systems implanted during the study period**. PECLA, pumpless extracorporeal lung assist; vv-ECMO, veno-venous extracorporeal membrane oxygenation.

During the study period we changed our ELS treatment regime due to developing more experience with miniaturized vv-ECMO in patients with respiratory failure (Figure 1). We did not use classic vv-ECMO in the phase between 2002 and 2008, since during that period available systems were characterized by high priming volumes and increased demand for anticoagulation, which we felt were not suitable in patients presenting with severe trauma. The advantage of PECLA use in these patients was lower anticoagulation and avoidance of stress to blood components by the pumpless technique. The choice of technique was not influenced by a specific indication, but by the specific supposed suitability of the device. After impressive technical development and miniaturization of vv-ECMO systems (centrifugal pump, low priming volume, smaller cannulas), we predominantly switched to such a technique. Therefore, since 2008 we primarily use vv-ECMO in trauma patients with ALF refractory to conventional treatment strategies for improvement of gas exchange.

ECMO provides sufficient extracorporeal oxygenation and removal of carbon dioxide. The extracorporeal device consisted of a membrane oxygenator and a centrifugal pump (Permanent Life Support System; Maquet Cardiopulmonary AG, Hirlingen/Rastatt, Germany). Since 2009, five patients were also provided with a single dual-lumen cannula inserted via the right jugular vein into the superior and inferior caval vein. The whole system is coated with heparin, hence a pronounced systemic anticoagulation is not necessary [13]. More details of vv-ECMO were already presented in previous studies from our working group [5,13,17]. Miniaturized veno-arterial ECMO was accomplished in patients with cardiopulmonary failure despite ELS in an emergency setting via the femoral vein and the femoral artery (*n *= 3). In all ELS devices, heparin-coated cannulas and circuits were used to decrease the systemic heparin dosage and to reduce post-traumatic bleeding complications [18].

### Patient management under extracorporeal lung support

After applying ELS, for patients with vv-ECMO the pump flow was adapted to ensure adequate oxygenation and to achieve protective mechanical ventilation (tidal volume ≤6 ml/kg predicted body weight, peak inspiratory pressure < 28 cmH_2_O). For patients under ELS mechanical ventilation, the parameters were reduced as soon as possible in order to perform lung-protective ventilation. For this purpose, FiO_2_, tidal volume and minute ventilation were stepwise decreased with the aim of a minimum PaO_2 _of 65 to 75 mmHg and a modest hypercapnia (partial pressure of arterial carbon dioxide 40 to 50 mmHg, except severe brain injury). Spontaneous breathing with assisted ventilation (positive airway pressure) was accomplished after 48 to 72 hours when clinical and radiological findings indicated the end of the vulnerable phase of acute lung injury. The mean arterial pressure was maintained between 60 and 75 mmHg by administering vasopressors continuously, especially in patients treated with PECLA. Intravenous heparin was applied to hold a partial thromboplastin time of about 50 to 60 seconds in PECLA patients without elevated bleeding risk. Our anticoagulation protocol used for vv-ECMO is also based on continuous heparin infusion, beginning with 600 IU/hour. We intend to reach a partial thromboplastin time of 40 to 50 seconds (control of partial thromboplastin time every 8 hours). This algorithm was individually modified with increasing hemorrhagic risk. In patients with high risk of further bleeding complications or evidence of relevant intracranial bleeding (Glasgow Coma Scale <9 and/or pathologic computed tomography scan), we avoided heparin for a certain period (≤48 hours). After bleeding control by repeated computed tomography scan, we slowly started to give heparin with a partial thromboplastin time range of 40-50 seconds.

Additional laboratory investigations included further coagulation and clotting parameters, hemoglobin and liver enzymes. In the early period (≤24 hours) arterial blood gases were drawn frequently (every 4 hours), while in the later period blood gases were analyzed every 8 hours. Basic monitoring of the lower extremities included continuous limb pulsoximetry distal to the arterial cannulation site, determination of serum lactate and creatinine kinase levels as well as clinical inspection for any signs of restricted perfusion and/or ischemia.

After respiratory and/or hemodynamic stabilization with adequate gas exchange, weaning from vv-ECMO was initiated by decreasing the extracorporeal blood flow to 1.5 l/minute. In addition, gas flow was scaled down (ECMO withdrawal trial). Afterward the system was switched off and if no marked deterioration of gas exchange and/or hemodynamics were observed within 2 hours, the ELS system was disconnected. Decannulation was achieved in most cases by simple manual compression of the vessel access site. Only in four patients was surgical removal of the cannulas (all arterial) necessary.

### Statistical analysis

Statistical analysis was performed with SPSS 16.0 (SPSS Inc., Chicago, IL, USA) for Windows (Microsoft Corp., Redmond, WA, USA) and Stata SE 10.1 for Windows (StatCorp., College Station, TX, USA). Descriptive statistics were used to describe patient characteristics throughout the study. Means and standard deviations were computed for normally distributed continuous variables, whereas medians and interquartile ranges (25th to 75th) were used to describe non-normally distributed continuous data. Student's *t *test was performed for comparison of normally distributed data and the Mann-Whitney U test was used for non-normally distributed data. Categorical variables are presented as frequency distributions (*n*) and simple percentages (%). Fisher's exact test was performed for categorical data in a 2×2 table or the chi-square test in a 2×3 table. *P *<0.05 was considered statistically significant.

## Results

### Demographic data

The study sample included 52 patients (49 male, three female) with a mean age of 32 ± 14 years (range, 16 to 73 years), with characteristics as presented in Table 1. Only trauma patients with concomitant chest injury were enrolled and all patients suffered from severe post-traumatic pulmonary failure that lead to consecutive hypercapnia and hypoxia refractory to conventional treatment strategies.

**Table 1:** Patient characteristics and different types of extracorporeal lung support

Variable	All patients (*n *= 52)	PECLA (*n *= 26)	vv-ECMO (*n *= 26)
Male gender	49 (94.2)	25 (96)	24 (92)
Age (years)	32 ± 14	34.5 ± 14.3	29.3 ± 13.2
Height (cm)	178.6 ± 7.2	179 ± 6.8	178.2 ± 7.6
Weight (kg)	89.8 ± 20.6	85.8 ± 16.5	93.8 ± 23.6
Body mass index (kg/m^2^)	28.2 ± 6.1	26.7 ± 4.5	29.6 ± 7.3
Trauma			
Traffic accident	38 (73)	17 (65)	21 (81)
Blast injury/gunshot wound	9 (17)	6 (23)	3 (11)
Deep fall	4 (8)	3 (12)	1 (4)
Blunt	1 (2)	0	1 (4)
Prior resuscitation	8 (15.4)	4 (15.4)	4 (15.4)
Acute renal failure prior to ELS	8 (15.4)	4 (15.4)	4 (15.4)
Injury Severity Score	58.9 ± 10.5	57.8 ± 10.9	59.4 ± 11.2
Lung Injury Score	3.3 ± 0.57	3.06 ± 0.65	3.53 ± 0.36
SOFA score	10.5 ± 3	9.2 ± 3	11.8 ± 2.4

Data presented as *n *(%) or mean ± standard deviation. ELS, extracorporeal lung support; PECLA, pumpless extracorporeal lung assist; SOFA, Sequential Organ Failure Assessment; vv-ECMO, veno-venous extracorporeal membrane oxygenation.

Major traffic accident was the most common trauma (73%), followed by blast injury/gunshot (17%), deep fall (8%), and blunt trauma (2%). All patients had multiple organ injuries. In 30 patients (58%) we observed moderate to severe head trauma. Within this subgroup, intracranial hematoma or bleeding was evident in 14 patients. The mean Injury Severity Score was 58.9 ± 10.5. Cardiopulmonary resuscitation was performed in 15% (*n *= 8) of all patients before implantation of the mechanical assist device. Acute renal failure requiring temporary renal replacement therapy was present in eight patients (15%) prior to ELS implantation. There were 40 (77%) patients with unilateral or bilateral serial/multiple rib fractures and concomitant hematothorax and pneumothorax. Additional post-traumatic or aspiration pneumonia was seen in 29 (56%) patients, causing the need for ELS support. The mean Lung Injury Score according to Murray was 3.3 ± 0.6 and the mean Sequential Organ Failure Assessment score was 10.5 ± 3.

### Changes of gas exchange and ventilation parameters

All relevant parameters prior to ELS implantation under maximal ventilator support are listed in Table 2 for all patients and also for each ELS system. The median PaO_2_/FiO_2 _ratio for all patients was 63 (49 to 101) and was comparably higher in patients treated with PECLA (97; 56 to 173) compared with patients treated with vv-ECMO (54; 48 to 65). No relevant differences were present regarding the median partial pressure of arterial carbon dioxide (68 mmHg vs. 67 mmHg). All patients had primary respiratory acidosis (median pH 7.25) due to hypercapnia.

**Table 2:** Gas exchange and ventilation parameters prior to extracorporeal lung support implantation

Variable	All patients (*n *= 52)	PECLA (*n *= 26)	vv-ECMO (*n *= 26)
PaO_2_/FiO_2_	63 (49 to 101)	97 (56 to 173)	54 (48 to 65)
PaO_2 _(mmHg)	63 (49 to 89)	80 (56 to 99)	54 (48 to 65)
PaCO_2 _(mmHg)	67 (50 to 87)	68 (50 to 84)	67 (49 to 97)
pH	7.23 (7.16 to 7.38)	7.25 (7.18 to 7.37)	7.21 (7.12 to 7.38)
Arterial oxygen saturation (%)	92 (79 to 97)	96 (83 to 98)	88 (74 to 93)
MV (l/minute)	12 (9 to 14)	12 (9 to 14)	11 (8 to 14)
V_T _(ml)	516 (438 to 567)	483 (430 to 560)	560 (458 to 613)
MAP (mmHg)	71 (65 to 81)	69 (65 to 80)	71 (65 to 81)
Lactate (mg/dl)	28 (14 to 49)	16 (10 to 30)	38 (21 to 70)

Data presented as median (25th to 75th interquartile range). FiO_2_, fraction of inspired oxygen; MAP, mean arterial pressure; MV, minute volume; V_T_, tidal volume; PaCO_2_, partial pressure of arterial carbon dioxide; PaO_2_, partial pressure of arterial oxygen; PECLA, pumpless extracorporeal lung assist; vv-ECMO, veno-venous extracorporeal membrane oxygenation.

**Figure 2:**
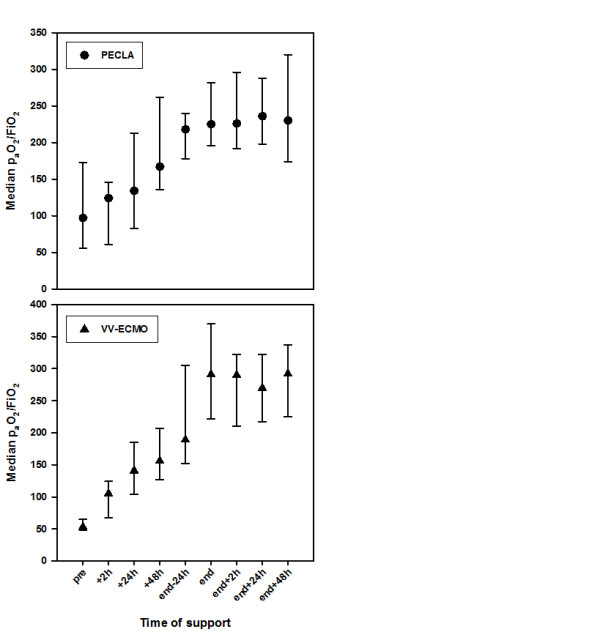
**Time course of the partial oxygen pressure/fraction of inspired oxygen ratio during extracorporeal lung support**. Time course of the partial oxygen pressure (PaO_2_)/fraction of inspired oxygen (FiO_2_) ratio during extracorporeal lung support separated into both systems. Median with 25th to 75th interquartile range. PECLA, pumpless extracorporeal lung assist; vv-ECMO, veno-venous extracorporeal membrane oxygenation.

**Figure 3:**
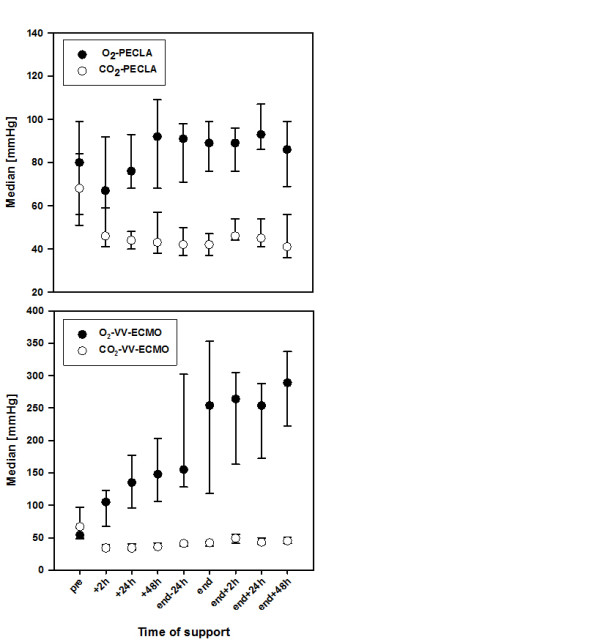
**Changes of relevant gas exchange parameters under extracorporeal lung support**. Median with 25th to 75th interquartile range. CO_2_, carbon dioxide; O_2_, oxygen; PECLA, pumpless extracorporeal lung assist; vv-ECMO, veno-venous extracorporeal membrane oxygenation.

**Figure 4:**
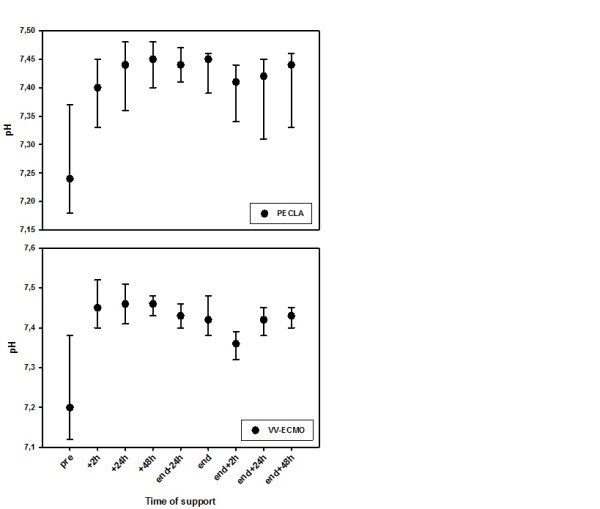
**Improvement and normalization of arterial pH immediately after extracorporeal lung support implantation in both groups**. Median with 25th to 75th interquartile range. PECLA, pumpless extracorporeal lung assist; vv-ECMO, veno-venous extracorporeal membrane oxygenation.

All data regarding gas exchange, ventilation and hemodynamic parameters in the course of time for PECLA as well as for vv-ECMO are shown in Table 3. In both systems the median PaO_2_/FiO_2 _ratio continuously increased immediately after initiating the ELS and stayed at an acceptable level until the ELS was removed (Figure 2). The median PaO_2 _values increased in patients treated with vv-ECMO, whereas there was only a mild increase in patients treated with PECLA (Figure 3). Otherwise, there was a decrease regarding the partial pressure of arterial carbon dioxide in the PECLA group due to effective carbon dioxide removal. These facts are mainly caused by the varying ELS systems used in the patients. In addition, before ELS implantation all patients had respiratory acidosis with a median pH of 7.25 (PECLA patients) and 7.21 (vv-ECMO patients), which rapidly improved to normal pH values directly after being supported by the extracorporeal device (Figure 4).

**Table 3:** Gas exchange, ventilation and hemodynamic parameters in the course of time for both ELS systems

Variable	Prior ELS implant	After 2 hours	1 day	2 days	ELS explant	1 day post
PECLA						
PaO_2_/FiO_2_	97 (56 to 173)	124 (61 to 147)	135 (83 to 213)	167 (136 to 263)	225 (196 to 283)	236 (198 to 289)
PaO_2 _(mmHg)	80 (56 to 99)	67 (59 to 92)	76 (68 to 93)	92 (68 to 109)	89 (76 to 99)	93 (68 to 107)
PaCO_2 _(mmHg)	68 (50 to 84)	46 (41 to 59)	44 (40 to 48)	43 (38 to 57)	42 (37 to 47)	45 (41 to 54)
pH	7.25 (7.18 to 7.37)	7.41 (7.33 to 7.45)	7.44 (7.36 to 7.49)	7.45 (7.4 to 7.48)	7.45 (7.39 to 7.46)	7.42 (7.31 to 7.45)
MV (l/minute)	12 (9 to 14)	8 (6 to 13)	7 (5 to 8)	7 (5 to 9)	9 (8 to 12)	11 (9 to 14)
MAP (mmHg)	69 (65 to 80)	77 (68 to 81)	80 (75 to 90)	85 (74 to 96)	79 (71 to 99)	78 (68 to 85)
vv-ECMO						
PaO_2_/FiO_2_	54 (48 to 65)	105 (67 to 124)	141 (104 to 186)	158 (127 to 207)	291 (223 to 370)	270 (218 to 323)
PaO_2 _(mmHg)	54 (48 to 65)	105 (67 to 123)	135 (96 to 177)	148 (106 to 203)	254 (118 to 353)	253 (173 to 288)
PaCO_2 _(mmHg)	67 (49 to 97)	34 (30 to 39)	34 (31 to 40)	36 (34 to 42)	42 (36 to 44)	43 (40 to 50)
pH	7.21 (7.12 to 7.38)	7.46 (7.4 to 7.52)	7.47 (7.41 to 7.51)	7.46 (7.43 to 7.48)	7.42 (7.38 to 7.46)	7.42 (7.38 to 7.45)
MV (l/minute)	11 (8 to 14)	6 (5 to 7)	4 (3 to 5)	5 (3 to 6)	8 (6 to 11)	12 (9 to 14)
MAP (mmHg)	71 (65 to 81)	79 (68 to 83)	77 (69 to 85)	76 (68 to 82)	76 (71 to 86)	72 (65 to 81)

Data presented as median (25th to 75th interquartile range). ELS, extracorporeal lung support; FiO_2_, fraction of inspired oxygen; MAP, mean arterial pressure; MV, minute volume; V_T_, tidal volume; PaCO_2_, partial pressure of arterial carbon dioxide; PaO_2_, partial pressure of arterial oxygen; PECLA, pumpless extracorporeal lung assist; vv-ECMO, veno-venous extracorporeal membrane oxygenation.

### Extracorporeal lung support implantation and management strategies

Data regarding ELS are presented in Table 4. All patients had mechanical ventilation before implantation of the ELS system with a mean time of 3.2 ± 4.1 days. Slightly more than one-half of the devices were implanted at the University Medical Center Regensburg (*n *= 27; 52%) as well as in outlying hospitals or the battle field (Iraq/Afghanistan) with ground or air transport to our hospital (*n *= 25; 48%). Almost 70% of all patients treated with vv-ECMO received ELS implantation in an external facility, in contrast to only 29% of patients treated with PECLA. All patients underwent peripheral cannulation. In 98% of patients, cannulation was performed via a percutaneous approach (Seldinger's technique). Only in one patient were both cannulas inserted through an open surgical access (PECLA). Ultrasonography was performed in all patients to ensure correct anatomic location prior to vessel puncture. Afterwards the guidewires were inserted and the cannulas were implanted.

**Table 4:** Implantation data, parameters during ELS, complications and outcome

Variable	All patients (*n *= 52)	PECLA (*n *= 26)	vv-ECMO (*n *= 26)
External ELS implantation	25 (48)	7 (26.9)	18 (69.2)
Percutaneous cannulation	51 (98)	25 (96)	26 (100)
Surgical cannula removal	4 (7.7)	4 (15.4)	0 (0)
Pre-ELS mechanical ventilation (days)	3.2 ± 4.1 (0 to 21)	3.7 ± 4.6 (0 to 21)	2.6 ± 3.6 (0 to 16)
Time from trauma to ELS (days)	5.2 ± 7.7 (0 to 38)	5.9 ± 8.1 (0 to 38)	4.5 ± 7.3 (0 to 34)
Duration of ELS (days)	6.9 ± 3.6 (<1 to 19)	7.6 ± 4 (<1 to 19)	6.3 ± 3.1 (<1 to 13)
Flow rate (l/minute)	2.3 ± 0.9 (0.7 to 4.6)	1.7 ± 0.5 (0.7 to 2.9)	3 ± 0.6 (1.8 to 4.6)
Duration of mechanical ventilation (days)	18.4 ± 10.6 (1 to 51)	18.7 ± 10.4 (1 to 49)	18.1 ± 11 (1 to 51)
Cannula-related complications	8 (15.4)	5 (19.2)	3 (11.5)
Renal-replacement therapy	16 (30.8)	9 (34.6)	7 (26.9)
ICU stay (days)	22 (14 to 32)	23 (18 to 35)	17 (13 to 30)
Hospital stay (days)	25 (16 to 41)	25 (21 to 39)	24 (13 to 44)
Surgical procedure	45 (86.5)	21 (80.8)	24 (92.3)
Thoracic surgical procedure	8 (15.4)	4 (15)	4 (15.4)
Surgery with ELS	16 (30.8)	6 (23.1)	10 (38.5)
Weaning from ELS	44 (84.6)	22 (85)	22 (84.6)
Death on ELS system	8 (15.4)	4 (15.4)	4 (15.4)
In-hospital mortality	11 (21.2)	6 (23.1)	5 (19.2)

Data presented as *n *(%),mean ± standard deviation (minimum to maximum), or median (25th to 75th interquartile range). ELS, extracorporeal lung support; PECLA, pumpless extracorporeal lung assist; vv-ECMO, veno-venous extracorporeal membrane oxygenation.

There were no severe system-related complications (cannula dislocation, ELS dysfunction) during intensive care transport. The mean time from trauma to installation of the extracorporeal device was 5.2 ± 7.7 days (median time 3 days) and varied from <24 hours up to 38 days. In all patients treated with PECLA, the femoral vein and the femoral artery were cannulated (median cannula size 17F; range, 15F to 19F). In 18 (69%) patients treated with vv-ECMO the venous drainage was obtained from the femoral and jugular vein, in two (8%) patients also from the subclavian vein or the contralateral femoral vein (*n *= 1; 4%). The median cannula size was 21F (range, 17F to 27F). The single double-lumen cannula (Avalon; Maquet, Rastatt, Germany) was used in five (19%) patients with a median cannula size of 23F (range, 23F to 27F). The mean pump flow was 3 ± 0.64 l/minute and was adjusted to allow protective ventilation and sufficient gas exchange.

Cannula-related complications, including mostly ischemia or minor bleeding, occurred in 15% of all patients and were more frequent in patients with arterial cannulation (PECLA, 19%) than in those with only venous cannulas (vv-ECMO, 12%). There was one patient with a need for lower limb fasciotomy on both sides due to venous femoral cannulation (left side), but there was no patient with a need for amputation due to venous or arterial cannulation. The overall median length of ICU stay was 22 days and the median stay in our university hospital was 25 days. Afterwards patients were discharged or transferred to another hospital or rehabilitation center.

Whenever necessary, further operative treatment was performed before, during or after ELS for life salvage. In total, surgery was performed in 45 (87%) patients, with 16 (31%) patients under ELS prevention. Nonthoracic surgical procedures included trauma surgery (that is, stabilization of limb fractures, spine surgery) and general surgery (that is, abdominal packing, splenectomy). In patients with intracranial bleeding (*n *= 14), 11 (79%) were treated with an external ventricular drainage and three patients (21%) also underwent neurosurgical craniotomy with evacuation of hematoma. Many patients had more than one operation during their hospitalization. In this series we found no relevant, life-threatening bleeding complications related to secondary nonthoracic surgery. There were 12 patients with abdominal bleeding, which could be successfully treated. In eight (15%) patients, extended thoracic surgical interventions (lung resection, active bleeding control) were also performed before or after ELS implantation. No relevant postoperative bleeding complications were noted. During ELS, the overall transfusion rate of packed red blood cells in all patients during ELS was of median 3 (range, 0 to 54), with no significant differences between PECLA (median 3; range, 0 to 12) and vv-ECMO (median 3; range, 0 to 54). Fresh frozen plasma was only needed in one patient treated with PECLA (*n *= 14 fresh frozen plasma units), whereas 10 patients with vv-ECMO required fresh frozen plasma (median 19 units; range, 1 to 102). One patient required a total of 54 packed red blood cell units, 102 fresh frozen plasma units and 11 platelet concentrates during vv-ECMO for 3 days. This patient died under ECMO support due to fulminant, diffuse bleeding.

The mean duration of ELS was 6.9 ± 3.6 days and was slightly, but insignificantly longer in patients treated with PECLA (7.6 ± 4 days) compared with patients treated with vv-ECMO (6.3 ± 3.1 days). Two patients with initial PECLA support were changed to veno-arterial ECMO because of progressive cardiopulmonary insufficiency. One patient treated with vv-ECMO developed cardiac failure, which mandated placement of veno-arterial ECMO on an emergency basis. All three patients died due to multiorgan failure. Weaning from ELS was successful in 84.6% of all patients. Overall in-hospital mortality was 21% (*n *= 11) with a slightly higher mortality rate in patients with PECLA (23%) than in patients with vv-ECMO (19%). Leading causes of death were multiorgan failure (*n *= 9), fulminant bleeding (*n *= 1) and cerebral hypoxia and bleeding with entrapment (*n *= 1). No death was a direct related result of the ELS support with respect to vessel cannulation or our anticoagulation regime.

## Discussion

The mortality of patients with acute respiratory failure remained high throughout recent years and was reported to be approximately 27 to 45% [19]. On the other hand, the ARDS mortality rate in both blunt and penetrating trauma patients decreased over time [20]. ECMO has the unique potential to support gas exchange without causing further lung damage from invasive positive pressure ventilation (barotrauma) in adult patients with fulminant respiratory failure and may improve patient survival [21]. ECMO may thus provide an additional treatment modality in patients with severe traumatic lung injury with ALF that does not respond to conventional treatment and ventilatory regimes [8]. Recently, ECMO therapy also presents a rescue therapy in severe trauma patients with concomitant chest injury suffering from refractory ALF when conventional therapies have been exhausted [2,18]. In previous reports, ELS devices have been safely used in adult trauma patients with multiple injuries and severe pulmonary failure with an improved survival after early implementation [9]. Quick encouragement for and short duration of ECMO for the temporary management of gas exchange has been reported to improve survival rates in trauma patients with ARDS [12]. However, to our knowledge in the recent literature there are only case reports or studies with limited numbers of patients.

In this report we describe our interdisciplinary experience with ELS including PECLA and vv-ECMO in severe thoracic trauma patients with ALF. In particular, this study sample deals with the largest number of patients who were treated with this special management of ELS therapy.

The main findings of our study are as follows. First, ELS enabled a rapid and sustained improvement of oxygenation, removal of carbon dioxide and correction of respiratory acidosis. In addition, our data demonstrate that ELS can provide advanced lung-protective ventilation strategies in patients suffering from severe post-traumatic ALF. Pulmonary recovery sufficient to wean the patient from ELS occurred in 85% of patients. Third, our overall hospital survival rate to discharge in these trauma patients with severe ALF was 79%, which is markedly better compared with Injury Severity Score-related mortality rates from the trauma databank (mean 59%).

Emergency thoracotomy due to fulminate bleeding had to be performed in nine patients before or after ELS implantation. Nevertheless, ECMO is a therapy with potentially serious complications [13]. In this study sample, no device-related complications including rupture of the circuits or relevant failure of the oxygenator were seen. In the past, bleeding disorders, especially in trauma patients, had been a major complication of ELS devices related to the requirement for systemic anticoagulation [22]. Further developments in ECMO systems and the improvement of anticoagulation management of the circuits (heparin coated) have led to decreased hemorrhagic complications [2,12]. Heparin-bond circuits therefore offer supplemental capability in the resuscitation of selected massively injured patients while their primary injuries have been evaluated and treated [23]. Miniaturized ECMO systems with improved oxygenators, circuits and centrifugal pumps have further markedly decreased hemorrhagic complications, which might make its implementation possible in patients with a higher risk of bleeding [13]. However, few data are available regarding ECMO in trauma patients with increased risk for bleeding complications. A recent case series recommends prolonged heparin-free vv-ECMO therapy in multiple traumatized patients with ALF with coexisting traumatic brain injury and intracranial bleeding. Neither ECMO-associated bleeding nor clotting of the extracorporeal circuit occurred and all three patients survived [24].

By using heparin-coated cannulas and circuits in addition to a lower systemic anticoagulation regime, the bleeding complication rate of our trauma patients was within an acceptable range. In addition, patients with coexisting post-traumatic bleeding were treated with initial heparin-free ECMO, as previously reported from our working group [18]. But less or no systemic heparin administration has a potential higher risk for thrombosis of the oxygenator. Accurate monitoring and contingent emergent change of the oxygenator might therefore be necessary. In this series we did not observe this drastic complication. Relevant bleeding complications at cannulation sites could be treated in most cases (*n *= 3) by gentle manual pressure and only in one patient was surgical correction necessary. Another serious complication of prolonged peripheral arterial cannulation remains ischemia of the limb [12,25]. Three patients (19%) with PECLA developed peripheral ischemia due to arterial cannula placement and required emergent surgical correction and switch of the cannulation site. The PECLA device with a heparin-coated oxygenation membrane provides sufficient blood flow without the need for an additional roller pump [14,26,27]. This device is commonly used for primary pulmonary stabilization in peripheral hospitals or even on the battlefield [27,28].

In the course of the study period we switched to vv-ECMO to further reduce the complication rate regarding ischemia or arterial bleeding. vv-ECMO has the advantage of avoiding arterial vessel complications, including ischemia. In trauma patients, arterial vessel access (femoral) is often limited whereas there are five locations for venous vessel access to place the ECMO. Only in 12% of our ECMO patients were bleeding complications due to venous cannulation recorded and all could be treated conservatively. Furthermore, ECMO can also be used in patients with hemodynamic instability. Adjustment of the ECMO pump flow provides significantly better oxygenation as well as elimination of carbon dioxide compared with PECLA. Finally, patients treated with ECMO presented with worse preimplantation data (higher Lung Injury Score and Sequential Organ Failure Assessment score), but their early outcome was slightly better (81%) compared with patients treated with PECLA (77%). However, due to a different physiologic concept (low flow of arterial blood through the membrane), PECLA is predominantly characterized by efficient carbon dioxide removal and moderate oxygenation improvement. In trauma patients with a leading hypercapnic lung failure and without severe hypoxemia, who additionally are suffering from bleeding complications, PECLA is a suitable alternative to ECMO since the anticoagulation demand is low.

This single-center study has some limitations. The main limitation of our study is its retrospective, nonrandomized design without a control group and the duration over a 10-year study period. This implies that conclusions are necessarily limited in their application and causality cannot be determined. Furthermore, we were not able to give data on long-term survival. Despite these limitations, we present a large number of severe trauma patients with ALF, who were interdisciplinarily treated with two different types of ELS in an experienced ECMO center. Finally, we present prospectively collected data from the Regensburg ECMO Registry.

## Conclusion

ELS devices are an excellent and life-saving treatment option in severe thoracic trauma patients with ALF. Thoracic trauma patients with concomitant refractory pulmonary failure have a remarkable potential to recover under ELS. The utilization of the ELS devices was safe and effective in these severe multiple trauma patients. Furthermore, we observed no significantly higher rates of bleeding complications during ELS. But ELS remains a highly specialized treatment option that is only available in a few centers and the optimal therapy is complex. An interdisciplinary treatment approach may facilitate the survival in an experienced trauma center. In our current strategy, we encourage the use of early vv-ECMO support due to reduced complication rates, better oxygenation and best short-term outcome in patients with severe post-traumatic ALF.

## Key messages

• ELS devices are a life-saving treatment option in severe thoracic trauma patients with ALF.

• ELS enabled a rapid and sustained improvement of oxygenation, removal of carbon dioxide and correction of respiratory acidosis.

• ELS could provide advanced lung-protective ventilation strategies in patients suffering from severe post-traumatic ALF. Pulmonary recovery sufficient to wean the patient from ELS occurred in 85% of patients.

• The overall hospital survival rate to discharge in these multiple trauma patients with severe ALF was 79%.

• Utilization of vv-ECMO support was associated with acceptable complication rates and provided sufficient oxygenation.

## Abbreviations

ALF: acute lung failure; ARDS: acute respiratory distress syndrome; ECMO: extracorporeal membrane oxygenation; ELS: extracorporeal lung support; FiO_2_: fraction of inspired oxygen; PaO_2_: partial pressure of arterial oxygen; PECLA: pumpless extracorporeal lung assist; vv-ECMO: veno-venous extracorporeal membrane oxygenation.

## Competing interests

The authors declare that they have no competing interests.

## Authors' contributions

MR made substantial contributions to the design of this study, acquisition of data, data analysis and interpretation of data, and wrote and revised the manuscript. TB made substantial contributions to the design of this study, and has been involved in drafting the manuscript and revising it critically for important intellectual content. AP, BG, TM, CS and DZ made substantial contributions to conception and design of this study, and have been involved in revising the manuscript critically for important intellectual content. CD made substantial contributions to the design of this study, helped with data analysis and interpretation of data as well as revising the manuscript critically for important intellectual content. H-SH made substantial contributions to the design of this study, and has been involved in drafting the manuscript and revising it critically for important intellectual content. All authors gave final approval of the version to be published.

## References

[B1] Vecsei V Arbes S Aldrian S Nau T Chest injuries in polytrauma Eur J Trauma 2005 17 239 243 10.1007/s00068-005-2033-9

[B2] Huang YK Liua KS Lua MS Wua MY Tsai FC Lina PJ Extracorporeal life support in post-traumatic respiratory distress patients Resuscitation 2009 17 535 539 10.1016/j.resuscitation.2009.02.016 19362409

[B3] Round JA Mellor AJ Anaesthetic and critical care management of thoracic injuries JR Army Med Corps 2010 17 139 149 10.1136/jramc-156-03-02 20919613

[B4] Michaels AJ Management of post traumatic respiratory failure Crit Care Clin 2004 17 83 99 10.1016/S0749-0704(03)00099-X 14979331

[B5] Schmid C Philipp A Hilker M Rupprecht L Arlt M Keyser A Lubnow M Mueller T Venovenous extracorporeal membrane oxygenation for acute lung failure in adults J Heart Lung Transpl 2012 17 9 15 10.1016/j.healun.2011.07.013 21885295

[B6] Keel M Meier C Chest injuries - what is new? Curr Opin Crit Care 2007 17 674 679 10.1097/MCC.0b013e3282f1fe71 17975389

[B7] Tsushima K King LS Aggarwal NR De Gorordo A D'Alessio FR Kubo K Acute lung injury review Intern Med 2009 17 621 630 10.2169/internalmedicine.48.1741 19420806

[B8] Cordell-Smith JA Roberts N Peek GJ Firmin RK Traumatic lung injury treated by extracorporeal membrane oxygenation (ECMO) Injury 2006 17 29 32 10.1016/j.injury.2005.03.027 16243331

[B9] Michaels AJ Schriener RJ Kolla S Awad SS Rich PB Reickert C Younger J Hirschl RB Bartlett RH Extracorporeal life support in pulmonary failure after trauma J Trauma 1999 17 638 645 10.1097/00005373-199904000-00013 10217227

[B10] Peek GJ Moore HM Moore N Sosnowski AW Firmin RK Extracorporeal membrane oxygenation for adult respiratory failure Chest 1997 17 759 764 10.1378/chest.112.3.759 9315812

[B11] Bein T Weber F Philipp A Prasser C Pfeifer M Schmid FX Butz B Birnbaum D Taeger K Schlitt HJ A new pumpless extracorporeal interventional lung assist in critical hypoxemia/hypercapnia Crit Care Med 2006 17 1372 1377 10.1097/01.CCM.0000215111.85483.BD 16540950

[B12] Madershahian N Wittwer T Strauch J Franke UF Wippermann J Kaluza M Whalers T Application of ECMO in multitrauma patients with ARDS as rescue therapy J Card Surg 2007 17 180 184 10.1111/j.1540-8191.2007.00381.x 17488410

[B13] Müller T Philipp A Luchner A Karagiannidis C Bein T Hilker M Rupprecht L Langgartner J Zimmermann M Arlt M Wenger J Schmid C Riegger GA Pfeifer M Lubnow M A new miniaturized system for extracorporeal membrane oxygenation in adult respiratory failure Crit Care 2009 17 R205 10.1186/cc8213 20017915 PMC2811933

[B14] Philipp A Behr R Reng M Kaiser M Birnbaum D Pumpless extracorporeal lung assist J Extra Corpor Technol 1998 17 38 41

[B15] Zimmermann M Bein T Arlt M Philipp A Rupprecht L Mueller T Lubnow M Graf BM Schlitt HJ Pumpless extracorporeal interventional lung assist in patients with acute respiratory distress syndrome: a prospective pilot study Crit Care 2009 17 R10 10.1186/cc7703 19183475 PMC2688123

[B16] Müller T Lubnow M Philipp A Bein T Jeron A Luchner A Rupprecht L Reng M Langgartner J Wrede CE Zimmermann M Birnbaum D Schmid C Riegger GA Pfeifer M Extracorporeal pumpless interventional lung assist in clinical practice: determinants of efficacy Eur Respir J 2009 17 551 558 10.1183/09031936.00123608 19010979

[B17] Camboni D Philipp A Lubnow M Bein T Haneya A. Diez C Schmid C Mueller T Support time-dependent outcome analysis for veno-venous extracorporeal membrane oxygenation Eur J Cardiothorac Surg 2011 17 1341 1347 21700473 10.1016/j.ejcts.2011.03.062

[B18] Arlt M Philipp A Voelkel S Rupprecht L Mueller T Hilker M Graf BM Schmid C Extracorporeal membrane oxygenation in severe trauma patients with bleeding shock Resuscitation 2010 17 804 809 10.1016/j.resuscitation.2010.02.020 20378236

[B19] ARDS Definition Task Force Ranieri VM Rubenfeld GD Thompson BT Ferguson ND Caldwell E Fan E Camporota L Slutsky AS Acute respiratory distress syndrome: the Berlin Definition JAMA 2012 17 2526 2533 22797452 10.1001/jama.2012.5669

[B20] Navarrete-Navarro P Rodriguez A Reynolds N West R Rivera R Scalea T Adult respiratory distress syndrome among blunt and penetrating trauma patients: demographics, mortality, and resource utilization over 8 years J Crit Care 2001 17 47 53 10.1053/jcrc.2001.25230 11481598

[B21] Peek GJ Elbourne D Mugford M Tiruvoipati R Wilson A Allen E Clemens F Firmin R Hardy P Hibbert C Jones N Killer H Thalanany M Truesdale A Randomised controlled trial and parallel economic evaluation of conventional ventilatory support versus extracorporeal membrane oxygenation for severe adult respiratory failure (CESAR) Health Technol Assess 2010 17 1 46 20642916 10.3310/hta14350

[B22] Voelckel W Wenzel V Rieger M Antretter H Padosch S Schobersberger W Temporary extracorporeal membrane oxygenation in the treatment of acute traumatic lung injury Can J Anaesth 1998 17 1097 1102 10.1007/BF03012399 10021960

[B23] Perchinsky MJ Long WB Hill JG Parsons JA Bennett JB Extracorporeal cardiopulmonary life support with heparin-bonded circuitry in the resuscitation of massively injured trauma patients Am J Surg 1995 17 488 491 10.1016/S0002-9610(99)80201-3 7747825

[B24] Muellenbach RM Kredel M Kunze E Kranke P Kuestermann J Brack A Gorski A Wunder C Roewer N Wurmb T Prolonged heparin-free extracorporeal membrane oxygenation in multiple injured acute respiratory distress syndrome patients with traumatic brain injury J Trauma Acute Care Surg 2012 17 1444 1447 22673280 10.1097/TA.0b013e31824d68e3

[B25] Bisdas T Beutel G Warnecke G Hoeper MM Kuehn C Haverich A Teebken OE Vascular complications in patients undergoing femoral cannulation for extracorporeal membrane oxygenation support Ann Thorac Surg 2011 17 626 631 10.1016/j.athoracsur.2011.02.018 21550582

[B26] Brederlau J Anetseder M Wagner R Roesner T Philipp A Greim C Roewer N Pumpless extracorporeal lung assist in severe blunt chest trauma J Cardiothorac Vasc Anaesth 2004 17 777 779 10.1053/j.jvca.2004.08.022 15650994

[B27] Bein T Osborn E Hofmann HS Zimmermann M Philipp A Schlitt HJ Graf BM Successful treatment of a severely injured soldier from Afghanistan with pumpless extracorporeal lung assist and neutrally adjusted ventilator support Int J Emerg Med 2010 17 177 179 10.1007/s12245-010-0192-x 21031042 PMC2926866

[B28] Bein T Zonies D Philipp A Zimmermann M Osborn EC Allan PF Nerlich M Graf BM Fang R Transportable extracorporeal lung support for rescue of severe respiratory failure in combat casualties J Trauma Acute Care Surg 2012 17 1450 1456 10.1097/TA.0b013e3182782480 23188237

